# NUDT21 inhibits bladder cancer progression through ANXA2 and LIMK2 by alternative polyadenylation

**DOI:** 10.7150/thno.36030

**Published:** 2019-09-23

**Authors:** Ming Xiong, Liang Chen, Lijie Zhou, Yong Ding, Gallina Kazobinka, Zhaohui Chen, Teng Hou

**Affiliations:** Department of Urology, Union Hospital, Tongji Medical College, Huazhong University of Science and Technology, Wuhan 430022, China.

**Keywords:** NUDT21, bladder cancer, Wnt/β-catenin, NF-κB

## Abstract

**Purpose:** Nudix Hydrolase 21 (NUDT21) is a crucial mediator involved in alternative polyadenylation (APA), and this molecule has been reported to be a tumor suppressor in human cancers. However, neither the role NUDT21 plays in bladder cancer (BC) nor the mechanisms which are involved have been investigated.

**Methods:** Expression levels of NUDT21 in BC were evaluated with real-time PCR, western blotting, and immunohistochemistry (IHC). *In vitro* and *in vivo* assays were performed to investigate the function of NUDT21 in tumorigenesis in bladder cancer cells. The TOP/FOP flash reporter assay, western blot, and global APA site profiling analysis were used to identify the pathway which mediates the biologic roles of NUDT21 in BC.

**Results:** NUDT21 expression is reduced in BC tissue and cells, and BC patients with lower NUDT21 expression have shorter overall and recurrent-free survival than patients with higher NUDT21 expression. NUDT21 ectopic expression or knockdown respectively profoundly inhibited or promoted the capacity of BC cells for proliferation, migration and invasion. We also identified a number of genes with shortened 3'UTRs through modulation of NUDT21 expression, and further characterized the NUDT21-regulated genes ANXA2 and LIMK2. We found NUDT21 modulates the expression of ANXA2 and LIMK2 in the Wnt/β-catenin and NF-κB signaling pathways.

**Conclusions:** These findings show NUDT21 plays a crucial role in BC progression, at least in part through ANXA2 and LIMK2 which act by alternative polyadenylation. NUDT21 may thus have potential as a diagnostic and therapeutic target in treatment of BC.

## Introduction

Bladder cancer (BC) is the 9th most common malignancy worldwide, and is a leading cause of cancer death, with 400,000 new cases diagnosed every year, and 165,000 deaths annually 1. Moreover there are 80,000 new cases and over 32,000 deaths from BC annually in China 2. Although 70-80% of BC patients have noninvasive disease at the time of diagnosis, 10-20% of patients will eventually develop invasive or metastatic disease 3. The presence of local-regional invasion or metastatic recurrence hinders the effective treatment of BC, and the 5-year survival rate of patients with advanced stage disease is only slightly greater than 20% 4. Investigation of the molecular mechanisms underlying the development and progression of BC in order to better identify novel therapeutic targets is therefore warranted.

It has been demonstrated that approximately 70% of known human genes have multiple poly(A) sites, resulting in transcript variants with different 3′untranslated region (3'UTR) sequences, and this phenomenon is known as alternative polyadenylation (APA) 5. APA is now recognized as an important post-transcriptional regulator, which can affect gene expression in multiple ways. For example, more than 50% of human genes encode multiple transcripts derived from APA 6. Using genome-wide analysis, APA was found to influence the proliferation and differentiation status of cells by alteration of lengths of 3'UTRs 7. In particular, fast-growing or de-differentiated cell populations are generally associated with shorter 3'UTRs, while cells at late developmental stages tend to have longer 3'UTRs 8. It has become increasingly evident that APA plays crucial roles in both normal development and in diseases including cancer 9, 10. The effect of APA on cancer transcriptomes may be cancer type specific, but this remains to be further investigated 11.

NUDT21 is a member of the Nudix family of hydrolases, and it possesses a highly conserved subunit of the cleavage factor Im (CFIm) complex that participates in the assembly of eukaryotic pre-mRNA 12. This subunit has a NUDIX hydrolase domain that acts like an authentic RNA-binding protein 13, enabling NUDT21 to influence APA site choice by binding to the proximal cleavage and polyadenylation site, and directing APA 14. As an APA-associated protein, NUDT21 is involved in certain important biologic regulatory processes 15. NUDT21 contributes to the control of cell fate determination in a critical way. Using transcription-factor-induced reprogramming as a screening assay, Brumbaugh et al. found NUDT21 suppression facilitates generation of induced pluripotent stem cells, and delays progenitor cell differentiation 16. Moreover, NUDT21 is linked to human cancer progression 17.

However, the role of NUDT21 in BC is unknown. In the study we found NUDT21 functions as a tumor suppressor gene in BC progression. Overexpression of NUDT21 inhibited BC growth, while NUDT21 knockdown promoted BC growth and metastasis *in vitro* and *in vivo*. In addition, we show NUDT21 modulates the Wnt/β-catenin and NF-κB signaling pathways through regulation of the 3'-UTR of Annexin A2 (ANXA2) and LIM Domain Kinase 2 (LIMK2). These results provide new insight into the function of NUDT21 in BC, and offer a new perspective for development of therapeutic strategies for BC.

## Methods

### Cell culture

Bladder cancer cell lines (5637, UM-UC-3, TCCSUP, T24, EJ, SCaBER, T24T, J82, SW780) and uroepithelial SV-HUC-1 cells were obtained from the Cell Bank of the Chinese Academy of Sciences (Shanghai, China). Primary cultures of normal bladder urothelial cells (NBUCs) were established from fresh specimens of patients as described previously 18. All cancer cell lines were cultured in RPMI 1640 medium supplemented with 10% fetal bovine serum (FBS) (Gibico, USA). SV-HUC-1 cells were grown in F12K medium supplemented with 10% FBS.

### Tissue specimens

This study was conducted on 10 fresh BC tissue specimens and corresponding adjacent non-tumorous tissue, as well as on 196 paraffin-embedded BC specimens, which were histopathologically and clinically diagnosed at Huazhong University of Science and Technology affiliated Union Hospital. Another cohort of ten fresh bladder cancer tissues obtained from Huazhong University of Science and Technology affiliated Union Hospital were evaluated with qRT-PCR, Western blot, and immunostaining. The staging of all cancers was assigned according to the American Joint Committee on Cancer (AJCC) classification system for TNM staging. Tumor grade was determined by the criteria of the World Health Organization/International Society of Urological Pathologists. None of the patients included in this study had received radiotherapy or chemotherapy prior to surgery. This study was approved by the Ethics Committee of Huazhong University of Science and Technology affiliated Union Hospital. Written informed consent was obtained from all patients.

### RNA extraction and quantitative real-time PCR (qRT-PCR)

Total RNA was extracted from cells and tissues using the Trizol (Invitrogen) kit according to the manufacturer's instructions, and was reverse transcribed using the RevertAid First Strand cDNA Synthesis Kit (Thermo Scientific, MA, USA). Real-time PCR was performed on a StepOne Plus real-time PCR system (Life Technologies, Carlsbad, CA). The 2-ΔΔCT method was applied for relative quantitation. GAPDH was used as an internal control. Independent experiments were done in triplicate. Primer sequences are provided in Supplementary Table 1.

### Western blotting (WB)

Cells and tissue samples were lysed in RIPA lysis buffer, and protein concentration was determined with a BCA Protein Assay Kit (Thermo Scientific). Cell/tissue lysates were separated on SDS-PAGE gels and transferred to a polyvinylidene difluoride (PVDF) membrane (Millipore, Eschborn, Germany). The membranes were blocked in 5% milk, and were then incubated with primary antibodies: anti-NUDT21 (Abcam, cat. no. ab183660), anti-ANXA2 (Abcam, cat. no. ab41803), anti-LIMK2 (Abcam, cat. no. ab97766), anti-β-catenin (Abcam, cat. no. ab22656), anti-p-IκBα, IκBα and anti-p-IKKβ, IKKβ, anti-p65, anti-p84 (Cell signaling, Boston, MA, USA), and anti-α-tubulin (Santa Cruz, cat. no. sc-5286).

### Immunohistochemical (IHC) staining

Immunostaining was performed on paraffin-embedded 4 μm sections, which were deparaffinized in xylene and rehydrated. Antigen retrieval was performed by submerging the sections in a 10 μmol/L citrate buffer solution (pH 6.0) for 10 minutes in a microwave oven. Slides were then treated with 3% hydrogen peroxide in methanol to quench the endogenous peroxidase activity, followed by incubation with 1% fish skin gelatin to block nonspecific binding. Sections were incubated with anti-NUDT21 (Abcam, cat. no. ab183660) and anti-Ki67 (Abcam, cat. no. ab92742), anti-LIMK2 (Proteintech, cat. no. 12350-1-AP), anti-ANXA2 (Proteintech, cat. no. 11256-1-AP), anti-β-Catenin (Proteintech, cat. no. 51067-2-AP), and anti-p65(Proteintech, cat. no. 10745-1-AP) antibodies overnight at 4 °C. After washing, sections were treated with prediluted secondary antibody (Thermo Fisher Scientific, MA), followed by incubation with streptavidin-horseradish peroxidase complex (Thermo Fisher Scientific), and immersion in 3,3ʹ-diaminobenzidine. Slides were counterstained with 10% Mayer's hematoxylin and mounted with aqueous mounting medium. The degree of immunostaining was examined and scored independently by two observers who were blinded to patient clinical data. According to the proportion of positively stained tumor cells, sections were scored as follows: 0 (no staining); 1 (weak staining = light yellow); 2 (moderate staining = yellow brown) and 3 (strong staining = brown). The percentage of positive cells was scored as 0, negative; 1, 10% or less; 2, 11-50%; 3, 51-80%; or 4, or 80% or more positive cells. A final score was obtained by multiplying the staining intensity score and the proportion of positive tumor cells. A final score of 0-3 was considered as low expression, and a final score of 4-12 was considered as high expression.

### EdU Labeling

Cells were incubated with 5-ethynyl-2′-deoxyuridine (EdU, RiboBio; R11053) for 2 h at 37°C, and treated with ApolloR reaction cocktail according to the manufacturer's instructions. Images were collected using fluorescent microscopy (Olympus, Japan).

### Cell counting kit-8 (CCK-8) assay

Cell proliferation was quantified with the CCK-8 assay according to the manufacturer's protocol. Briefly, 5 × 10^3^ cells were seeded per well into a 96-well plate and incubated at 37°C. After incubation with 10 μL of CCK-8 reagent (DOJINDO Laboratories, Kumamoto, Japan) for 2 h, the optical density was measured at 450 nm using a microtiter plate reader. All experiments were performed in triplicate.

### Wound healing assay

Cells were seeded in 12-well plates and grown under permissive conditions until 90% confluence was reached. After a linear wound was created in the confluent monolayer using a pipette tip, cells were incubated 36 h in serum-free medium. The percentage decreases in the wound gaps were calculated using Image J software.

### Migration and invasion assays

The capacity for cell migration and invasion were evaluated using Transwell chambers (Corning LifeSciences). After pretreatment, 4 × 10^4^ cells suspended in 200 μL of serum-free medium were seeded in the upper chamber of the Transwell system, and medium supplemented with 10% FBS was added to the lower chamber. For the invasion assay, cells were seeded into 24-well plates with Matrigel-coated Transwell inserts. After incubation for 24 h, non-invasive or non-migrating cells remaining on the top surface were gently removed with a cotton swab. Cells which had migrated to the lower surface of the insert were fixed and stained with 0.1% crystal violet, and counted under a light microscope.

### Plasmids, lentiviral infection, and transfection

Human NUDT21 cDNA was amplified with PCR and cloned into a GV358 lentiviral vector (GeneChem, Shanghai, China). Oligos of NUDT21 shRNAs were synthesized and inserted into a GV248 vector Genechem Co., Ltd (GeneChem, Shanghai, China). Stable cell lines that expressed NUDT21 (NUDT21) or NUDT21-shRNAs (NUDT21-Ri1 and NUDT21- Ri2) were selected for 10 days with 0.5mg/ml puromycin. Three independent experiments were performed and the data are presented as mean ± SD.

### Luciferase reporter assay

Cells were seeded in triplicate wells of 24-well plates and allowed to settle for 12 hours. LEF/TCF and NF-ĸB luciferase reporter plasmids were cotransfected using Lipofectamine 2000 (Invitrogen, Carlsbad, CA, USA) 19 and cultured for 48 h, after which the cells were harvested and lysed for evaluation of luminescence using the Luciferase Reporter Assay Kit (Promega, Madison, WI, USA) according to manufacturer's instructions.

### 3'-RACE analysis

3'-RACE was performed to assess the 3'-end of ANXA2 and LIMIK2 mRNA sequences. Total RNA was reverse transcribed to cDNA by using the primer 5'-CCAGTGAGCAGAGTGACGAGGACTCGAGCTCAAGCTTTTTTTTTTTTTTTTT-3'. Primers specific for the proximal and distal poly(A) tails of the 3'-UTR were designed (Supplementary table 1) and mixed with the reverse primer: 5'-CCAGTGAGCAGAGTGACG-3'. The primer mixture, cDNA and Taq polymerase were combined to perform PCR under standard conditions. RACE PCR products were separated on a 1% agarose gel, and individual bands were gel purified and sequenced completely.

### Xenografted tumor model

Animal studies were approved by the Ethics Committee of Tongji Medical College of Huazhong University of Science and Technology. BALB/c-nu mice (5-6 wks of age) were purchased from the Center of Experimental Animals of Tongji Medical College of Huazhong University of Science and Technology, and were randomly divided into groups (n = 5/group). For the tumor formation assay, 3×10^6^ cells were injected subcutaneously into one side of each mouse. Tumor volumes were measured using an external caliper and calculated using the equation: (L × W^2^)/2. On day 30, tumors were evaluated with an IVIS imagining system (Caliper), and animals were euthanized. Tumors were then excised and subjected to pathologic examination. The *In Vivo* Optical Imaging System (*In Vivo* FX PRO, Bruker Corporation) was used to acquire fluorescent images of xenografts in nude mice.

### Statistical analysis

All statistical analysis was carried out using SPSS 16.0 software. Differences between two groups were analyzed with the non-parametric Mann-Whitney test. Comparisons between multiple groups were made by one-way analysis of variance (ANOVA) followed by Kruskal-Wallis ANOVA. Correlation between NUDT21 expression and clinicopathologic characteristics was analyzed using the chi-square test or Fisher's exact test. Survival curves were plotted by the Kaplan-Meier method and compared with the log-rank test. Data from at least 3 independent experiments are expressed as mean ± SD. P <0.05 was considered statistically significant.

## Results

### Decreased expression of NUDT21 is associated with aggressiveness in human BC

**Figure 1:**
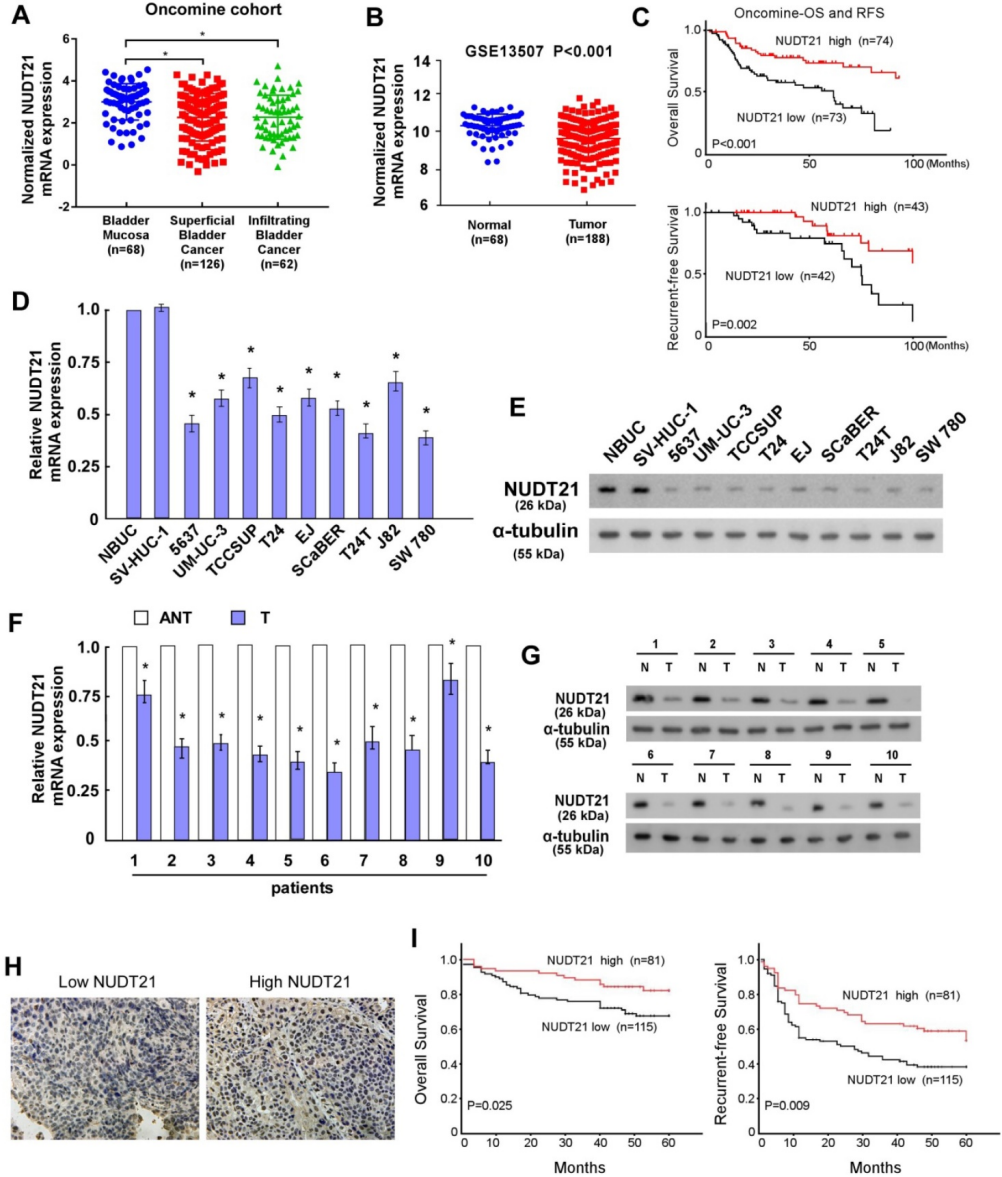
** NUDT21 expression is downregulated in bladder cancer and is correlated with prognosis.** (A-B) NUDT21 expression in Oncomine and GSE13507 cohorts (*P<0.001). (C) Overall and recurrence-free survival in the Oncomine BC dataset with low versus high levels of NUDT21 mRNA. The cut-point of NUDT21 mRNA expression was defined as the median. (D-E) NUDT21 mRNA and protein expression in 10 bladder cancers (T) and paired adjacent non-tumor tissues (ANT). (F-G) NUDT21 mRNA and protein expression in normal bladder urothelial cells (NBUCs), human uroepithelial cells (SV-HUC-1), and bladder cancer cell lines (5637, UM-UC-3, TCCSUP, T24, EJ, ScaBER, T24T, J82, and SW780). (H) Representative IHC analysis of NUDT21 in bladder cancer specimens. 400X. (I) Overall and recurrence-free survival curves in relation to NUDT21 status in 196 bladder cancer patients.

To explore the role of NUDT21 in BC, we initially investigated NUDT21 expression levels in BC. Expression data from two independent BC cohorts (Oncomine and GEO accession number GSE13507) were employed for validation. Statistical analysis showed that NUDT21 expression was decreased in BC tissues in these two cohorts (P < 0.05 for both cohorts; Figure 1A, 1B). It is of note that NUDT21 mRNA expression was negatively associated with overall and recurrent-free survival (P<0.001 and P=0.002 respectively, Figure 1C). We then assessed NUDT21 expression in 10 bladder cancer tissue specimens and cell lines with qRT-PCR and WB. NUDT21 expression was lower in tumors than in adjacent noncancerous tissues (Figure 1D, 1E). In addition, NUDT21 was significantly down-regulated in all 9 bladder cancer cell lines as compared with NBUCs and normal urothelial cells (Figure 1F, 1G).

To further evaluate the relationship of NUDT21 expression and the clinicopathologic features of BC, we performed IHC assays to assess NUDT21 expression in 196 archival formalin-fixed, paraffin-embedded human BC tissue samples (Figure 1H). This showed expression levels of NUDT21 were negatively correlated with tumor grade (P = 0.014), tumor size (P = 0.024), and clinical stage (T classification) (P = 0.016) (Supplementary table 2). In agreement with public BC data, Kaplan-Meier analysis revealed that NUDT21 expression is negatively associated with overall and recurrence-free survival (P=0.025 and P=0.009 respectively; Figure 1I). Moreover, multivariate analysis showed that NUDT21 expression is an independent prognostic factor for patients with BC (Supplementary table 3).

### NUDT21 suppresses cell proliferation, migration, and invasion *in vitro,* and suppresses tumor growth *in vivo*

**Figure 2:**
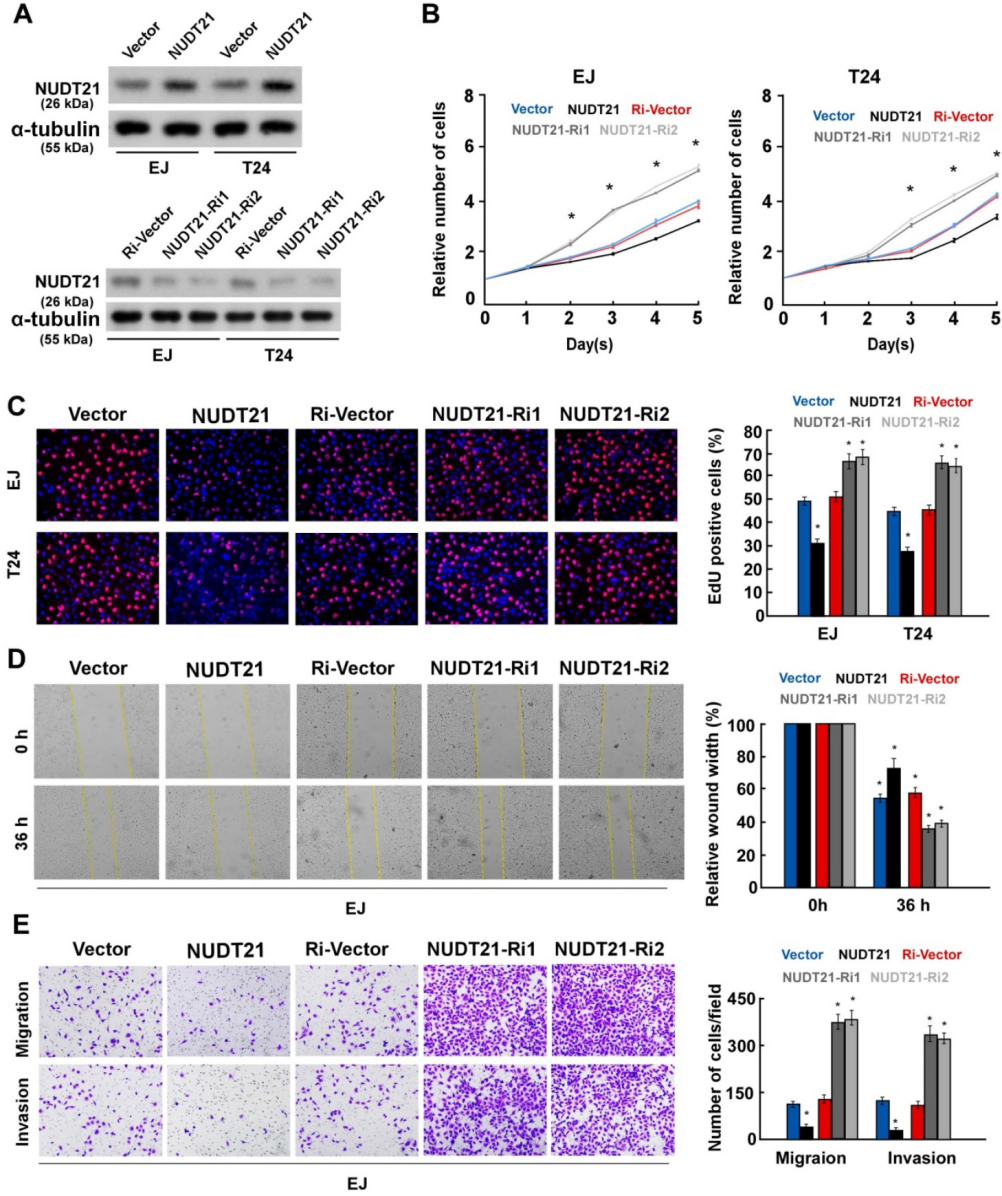
** NUDT21 inhibits aggressiveness of bladder cancer cells *in vitro*.** (A) Western blotting analysis of NUDT21 expression in EJ and T24 cells which stably express and silence NUDT21; α-tubulin was used as a loading control. (B) NUDT21 suppresses proliferation in bladder cancer cells, as determined by the CCK-8 assay. (C) Representative micrographs (left panel) and quantification (right panel) of EdU incorporation in bladder cancer cells as indicated. DAPI was used as a DNA/nuclear stain. (D) Mobility of cells was measured with the wound healing assay (left panels); uncovered areas in the wound healing assays were quantified as a percentage of the original wound area (right panels). (E) Representative pictures (left panel) and quantification (right panel) of invading cells as analyzed with a transwell Matrigel assay.

To investigate the biological role of NUDT21 in BC, we performed gain-of-function assays by transfecting NUDT21 overexpressing or interference vectors into EJ and T24 cells (Figure 2A). Ectopic expression of NUDT21inhibited cell proliferation, while silencing of NUDT21 expression promoted cell proliferation in both EJ and T24 cells (Figure 2B, 2C). Our results also indicate that NUDT21-transduced cells exhibited significantly decreased ability for invasion, while suppressing NUDT21 had the opposite effect (Figure 2D, 2E). These *in vitro* results further supported the concept NUDT21 suppresses aggressiveness in BC cells.

**Figure 3:**
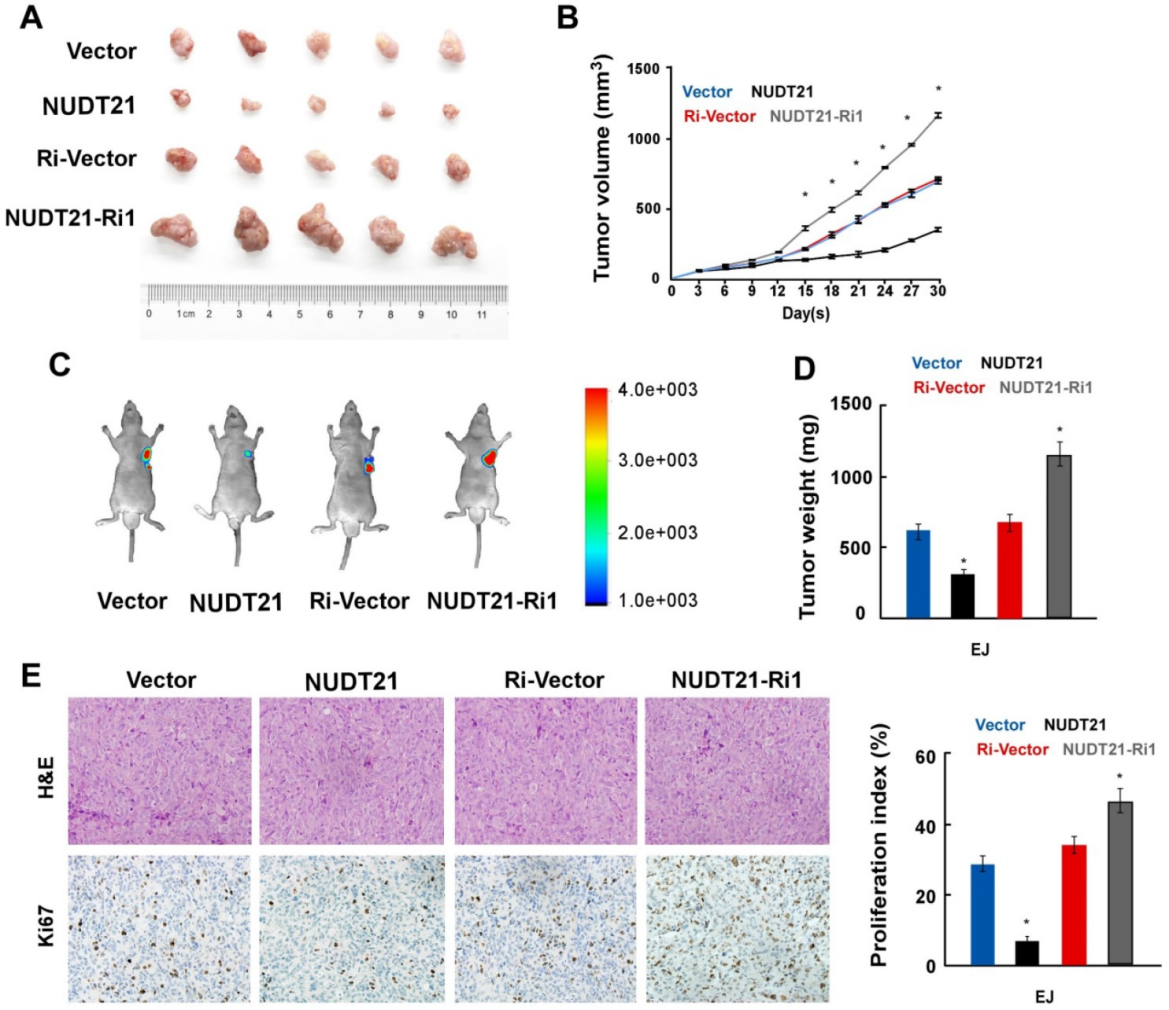
** NUDT21 suppresses tumorigenicity of bladder cancer *in vivo*.** (A) Representative images of tumor from a Xenograft model in nude mice. (B) Tumor volumes were measured on the indicated days. (C) Tumor volume monitored using the *In Vivo* Optical Imaging System. (D) Tumor weight of all mice in each group. (E) Immunohistochemical staining demonstrated expression of Ki67 in tissues as indicated. Data are represented as mean ± SD of three independent experiments. *P < 0.05.

To explore the impact of NUDT21 on BC growth *in vivo*, we implanted BC cells stably expressing NUDT21 or NUDT21-Ri vectors into the flanks of NOD/SCID mice subcutaneously. *In vivo* study showed that tumors overexpressing NUDT21 were significantly smaller than, and weighed less than control (Figure 3A-3D). Immunohistochemistry staining showed that NUDT21 significantly suppressed the expression of Ki67 (Figure 3E). These results indicate NUDT21 plays a significant role in suppressing BC tumorigenicity *in vivo*.

### NUDT21 regulates the length of the 3'-UTR of mRNA in BC cells

**Figure 4:**
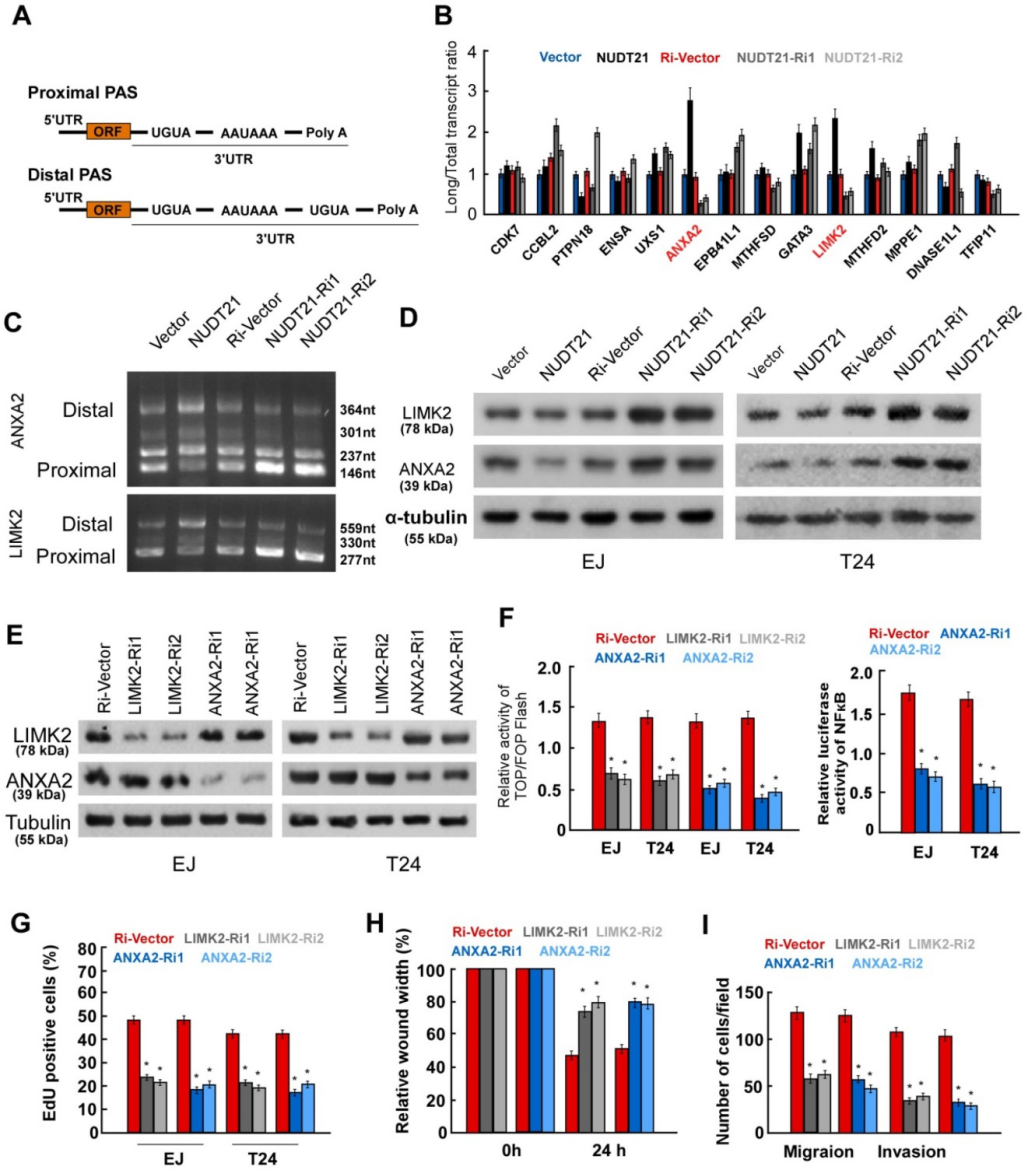
** NUDT21 regulates the length of the 3'-UTR of LIMK2 and ANXA2 genes.** (A) Schematic diagram of the 3'-UTR sequences in the model genes. (B) Transcripts with 3'-UTRs of different lengths were identified by real-time qPCR in NUDT21 overexpressing or knockdown cells, and the long transcript-to-total transcript ratios are shown. (C) Abundance of transcripts of different lengths identified by 3'-RACE in each cell line. More transcripts with longer 3'-UTRs were found in NUDT21 overexpressing cells, while more transcripts with shorter 3'-UTRs were found in NUDT21 knockdown cells. (D) Protein levels of LIMK2 and ANXA2 assessed in cells as indicated with western blotting. (E) Efficiency of shRNA targeting of LIMK2 and ANXA2 was evaluated with western blotting. (F) Analysis of TOP/FOP and NF-κB luciferase reporter activity in cells as indicated. (G) Quantification of cells as indicated in the EdU incorporation assay. (H-I) Quantification of invading cells analyzed using wound healing and transwell Matrigel assays. *P<0.05

To determine how NUDT21 contributes to APA and gene silencing in BC, we analyzed the composition of the 3′UTR sequences of specific oncogenes. We implemented a model that was previously used in hepatocellular carcinoma 20 to identify the NUDT21 responsive genes in BC. The following gene inclusion criteria were applied: (1) genes containing no less than two 3′UTR transcripts with the same starting point but different ending points, or (2) genes containing a poly A tail signal sequence (PAS: AAUAAA or a similar sequence) located 10-60 nt upstream of the 3′UTR, or (3) genes featuring proximal PASs within their long 3′UTR transcripts that were surrounded by 2 UGUA sequences located within 200 nt of the PASs (Figure 4A). In total, 165 genes in the Ensembl Human Genome Database met the criteria for inclusion in the model, and 49 were highly expressed in BC according to the oncomine database. Of these genes, 14 oncogenes were chosen for subsequent real-time PCR assay, in which primer pairs specific for both the long and the short transcripts of the 14 genes were used. We then calculated the long transcript-to-total transcript ratio for each gene, and selected genes for which the expression of long transcripts increased in NUDT21-overexpressing BC cells, and decreased in the NUDT21-knockdown cells (Figure 4B). Two genes, namely ANXA2 and LIMK2, fit our filtering criteria. To confirm ANXA2 and LIMK2 are subject to NUDT21-mediated APA, we performed 3′ RACE PCR analysis and identified PCR products of different lengths with direct DNA sequencing (Supplementary Figure 1-3). It is of note that most of the predicted miRNA binding sites localized to the region between the PAS1 and the distal APA site on the ANXA2 and LIMK2 3′UTRs (Supplementary Figure 4). The results showed that mRNA preferred distal cleavage sites and more transcripts with longer 3′-UTRs were generated in NUDT21-overexpressing cells. In contrast, mRNA preferred proximal cleavage sites and more transcripts with shorter 3′-UTRs were generated in NUDT21-knockdown cells (Figure 4C). Moreover, WB analysis revealed that ANXA2 and LIMK2 are downregulated in NUDT21-transfected cells, but are upregulated in NUDT21-knockdown cells (Figure 4D). These observations argue that expression of ANXA2 and LIMK2 are modulated by NUDT21 in BC.

ANXA2 and LIMK2 are known to play oncogenic roles in BC 21, 22, but the underlying mechanism is incompletely understood. To explore the mechanism by which ANXA2 and LIMK2 modify the aggressiveness of BC cells, we transfected shRNAs targeting ANXA2 and LIMK2 into BC cells (Figure 4E). Luciferase reporter assays showed that silencing of ANXA2 inhibited the activity of both Wnt/β-catenin and NF-κB signaling, while inhibition of LIMK2 suppressed Wnt/β-catenin signaling (Figure 4F). Moreover, silencing of either ANXA2 or LIMK2 drastically suppressed proliferation, migration, and invasion in both EJ and T24 cell lines (Figure 4G-4I). The above results strongly suggest ANXA2 and LIMK2 can activate Wnt/β-catenin and NF-κB signaling and exert oncogenic roles in BC cells.

### NUDT21 modulates Wnt and NF-κB signaling in BC cells

**Figure 5:**
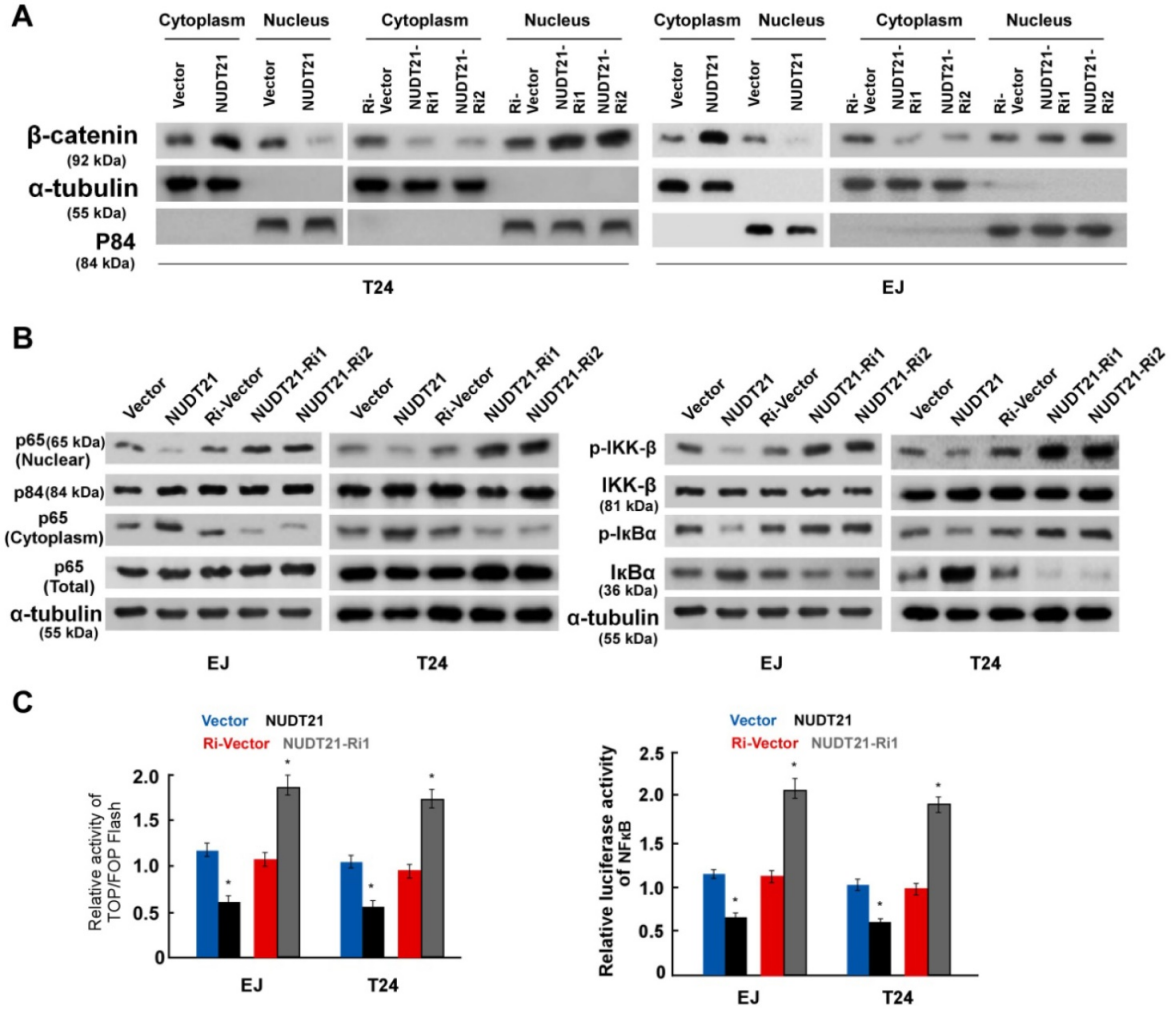
** NUDT21 activates the Wnt/β-catenin and NF-κB signaling pathways.** (A) Western blotting analysis of β-catenin expression in the nucleus and cytoplasm of cells as indicated. α-Tubulin and p84 were used as loading controls for the cytoplasmic and nuclear fractions respectively. (B) Western blotting analysis of protein expression levels in indicated cells. α-tubulin was used as a loading control. (C) Analysis of TOP/FOP and NF-κB luciferase reporter activity in cells as indicated. Bar graphs show statistical analysis for three independent experiments (* P<0.05).

To further evaluate the involvement of NUDT21 in the Wnt/β-catenin signaling pathway, we performed WB assays to examine the subcellular localization of β-catenin. Ectopic expression of NUDT21 resulted in decreased expression of β-catenin in the nucleus, while nuclear expression of β-catenin was increased in NUDT21-inhibited cells as shown in Figure 5A. Similarly, we examined the subcellular localization of P65 and the expression of phosphorylated-IKKβ and p-IκBα. As expected, overexpression of NUDT21 strongly inhibited expression of nuclear p65, p-IKKβ and p-IκBα (Figure 5B). Consistent with the changes observed in the WB assay, NUDT21 overexpression suppressed the activity of both β-catenin/TCF and NF-κB luciferase (Figure 5C). Based on these findings, we propose there is a pathway with NUDT21 mediated regulation of Wnt and NF-κB signaling which operates through ANXA2 and LIMK2 in BC cells.

### NUDT21 expression correlates with Wnt/β-catenin and NF-κB signaling pathways activation in human BC

**Figure 6:**
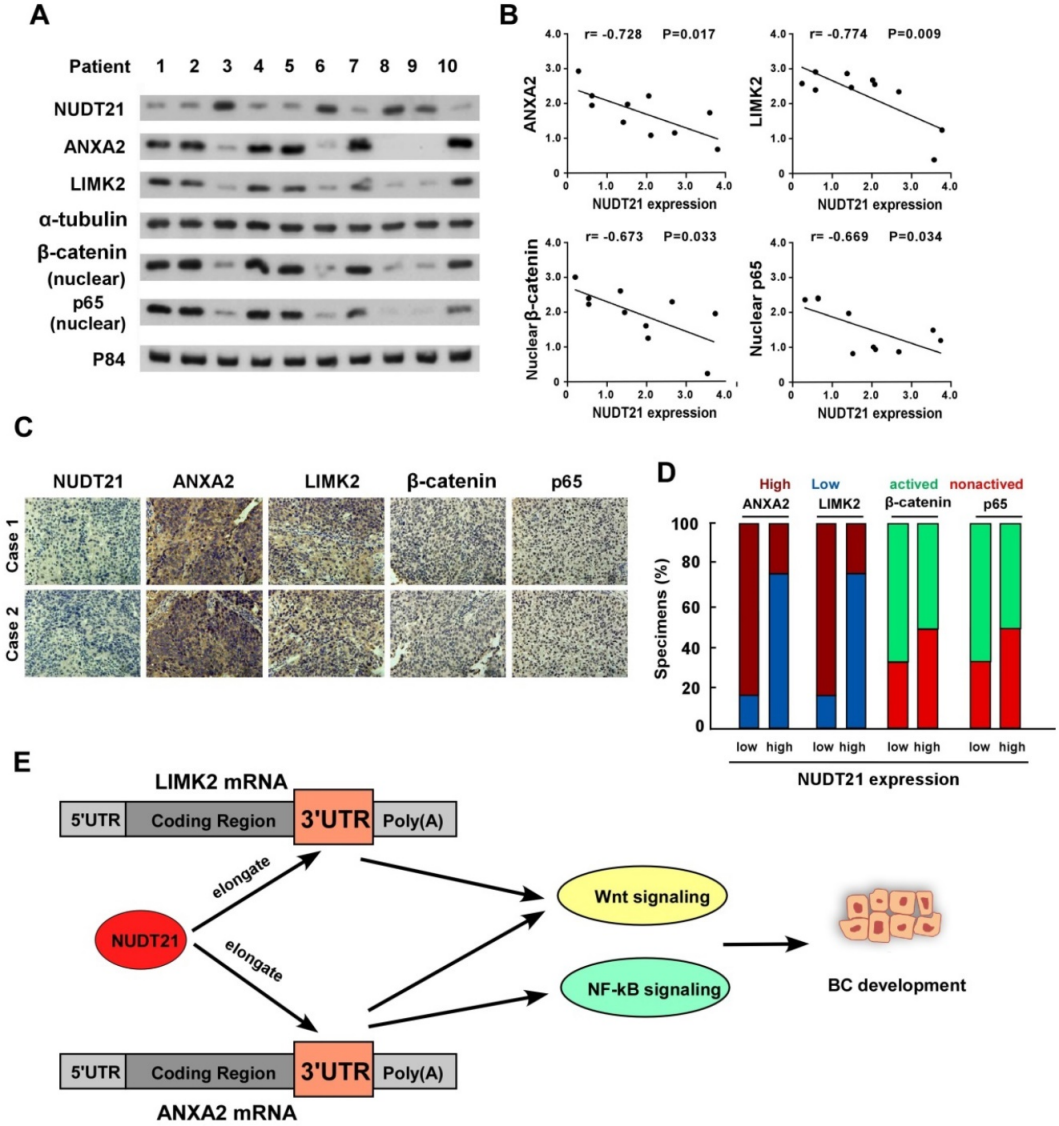
** Clinical relevance of NUDT21 mediation of Wnt/β-catenin and NF-κB signaling in bladder cancer.** (A) Western blot assay showing protein expression of NUDT21, LIMK2, ANXA2, nuclear β-catenin, and nuclear P65 expression in 10 bladder cancer tissue samples. α-Tubulin and p84 were used as loading controls. (B) Correlation analysis of NUDT21 and LIMK2, ANXA2, nuclear β-catenin and nuclear P65 expression respectively. (C) LIMK2, ANXA2, nuclear β-catenin, and nuclear P65 expression in two representative cases from these 10 bladder cancer samples. 400X. (D) Percentages of the 10 BC specimens showing the correlation of LIMK2, ANXA2, nuclear β-catenin, and nuclear P65 relative to low/high NUDT21 expression. (E) Schematic illustration of the model of the mechanism for the role of NUDT21in bladder cancer.

We next sought to determine whether the NUDT21-ANXA2/LIMK2-Wnt/NF-κB axis identified in BC cells is clinically relevant using 10 freshly prepared bladder cancer tissues. In these tissues, NUDT21 protein expression was negatively correlated with levels of ANXA2 (r = -0.728, p = 0.017), LIMK2 (r = -0.774, p = 0.009), nuclear β-catenin (r = -0.673, p = 0.033), and nuclear P65 (r = -0.669, p = 0.034) (Figure 6A-6B). These observations were validated using IHC analysis, in which NUDT21 expression showed negative correlation with the expression of ANXA2, LIMK2, activated β-catenin and P65 (Figure 6C-6D). Our results indicate downregulation of NUDT21 in BC is correlated with ANXA2 and LIMK2 expression, which in turn activates the Wnt/β-catenin and NF-κB signaling pathways to enhance the tumorigenicity of BC cells (Figure 6E).

## Discussion

The critical roles which 3′ UTRs plays in cancer development have previously been described, however the regulators, targets and impact of 3′ UTR-based regulation in BC pathogenesis has not been sufficiently studied. The results of the present study for the first time provide novel insight into the crucial role played by NUDT21 in regulation of APA-mediated 3′-UTR alterations, which further promote BC progression. Here we report NUDT21 inhibits cell proliferation, migration, and invasion, and represses tumorigenicity in BC. We characterized NUDT21-regulated genes with shortened 3′-UTRs, and found that ANXA2 and LIMK2 contribute to NUDT21-mediated tumor suppression by augmenting Wnt and NF-κB signaling. These findings identify a novel mechanism by which oncogene 3′-UTRs are regulated in BC, and suggest NUDT21 may be a potential target for BC therapy.

Aberrant expression of NUDT21 acts in the development and progression of human malignancies. For example, Tan et al. demonstrated NUDT21 inhibits hepatocellular carcinoma (HCC) proliferation, metastasis and tumorigenesis, in part by suppressing PSMB2 and CXXC5 17. In addition, Sun et al. reported that loss of NUDT21 shortens the 3′-UTR of various oncogenes (mainly RAB3IP, TMEM267, UBA5, and CCT5) in HCC cells, leading to unregulated tumor cell proliferation 20. In hematologic malignancy, silencing NUDT21 inhibits proliferation and promotes apoptosis of human K562 leukemia cells through regulation of p-ERK expression 23. These studies indicate the mechanism underlying NUDT21-mediated tumor suppression may be cancer specific and have different features in different malignancies. In the current study, we found upregulation of NUDT21 suppresses the proliferation, migration, and invasion of BC cells, while silencing of NUDT21 promotes these aggressive features in BC cells. NUDT21 inhibits BC aggressiveness by regulating the length of the 3′-UTRs of ANXA2 and LIMK2, and these genes have been found to be involved in the activation of the Wnt/β-catenin and NF-κB signaling pathways. Our findings therefore provide insight into the mechanism by which NUDT21 exerts tumor-suppression function in BC.

ANXA2 is an annexin family member, and has been found to interact with various ligands, and moreover is involved in the development of diverse cancers 24. Moreover, ANXA2 mediates the activation of both NF-κB and β-catenin 25, 26. In recent years, it has been found ANXA2 plays critical roles in bladder cancer formation, progression, and recurrence. However, the cellular and molecular mechanisms of ANXA2-mediated BC progression and therapeutic resistance have to date been incompletely studied 22, 27. At the same time, LIMK2 has been shown to act as an oncogene in bladder cancer, and is associated with the accumulation of β-catenin in the nucleus 28. In this study, we show ANXA2 and LIMK2 play oncogenic roles in mediation of the aggressiveness of bladder cancer cells. We further demonstrate NUDT21 elongates the 3′-UTRs of ANXA2 and LIMK2 genes, which consequently reduces their expression, and results in suppression of activation of the NF-κB and Wnt/β-catenin signaling pathways. The present study for the first time revealed NUDT21 not only attenuates Wnt/β-catenin signaling, but also inactivates NF-KB signaling in bladder cancer cells. Our results are supported by Lou and colleagues, who demonstrated NUDT21 is an upstream regulator of the NF-κB pathway which controls the mesenchymal identity of glioblastoma cells 29. Our data reveal a novel link between NUDT21 and signaling pathways during cancer development and progression.

## Conclusion

The current study for the first time reveals the correlation of NUDT21 mediated BC progression and APA. In addition, we show NUDT21 exerts its action through the Wnt/β-catenin and NF-κB signaling pathways. The sketch map shown in Figure 6E illustrates the mechanism of NUDT21 stimulated BC progression. NUDT21 has potential to serve as a biomarker and a potential therapeutic target for the treatment of BC.

## References

[1] Antoni S, Ferlay J, Soerjomataram I, Znaor A, Jemal A, Bray F (2017). Bladder Cancer Incidence and Mortality: A Global Overview and Recent Trends. Eur Urol.

[2] Chen W, Zheng R, Baade PD, Zhang S, Zeng H, Bray F (2016). Cancer statistics in China, 2015. CA Cancer J Clin.

[3] Abufaraj M, Dalbagni G, Daneshmand S, Horenblas S, Kamat AM, Kanzaki R (2018). The Role of Surgery in Metastatic Bladder Cancer: A Systematic Review. Eur Urol.

[4] Park JC, Citrin DE, Agarwal PK, Apolo AB (2014). Multimodal management of muscle-invasive bladder cancer. Curr Probl Cancer.

[5] Dalziel M, Nunes NM, Furger A (2007). Two G-rich regulatory elements located adjacent to and 440 nucleotides downstream of the core poly(A) site of the intronless melanocortin receptor 1 gene are critical for efficient 3' end processing. Mol Cell Biol.

[6] Tian B, Hu J, Zhang H, Lutz CS (2005). A large-scale analysis of mRNA polyadenylation of human and mouse genes. Nucleic Acids Res.

[7] Di Giammartino DC, Nishida K, Manley JL (2011). Mechanisms and consequences of alternative polyadenylation. Mol Cell.

[8] Sandberg R, Neilson JR, Sarma A, Sharp PA, Burge CB (2008). Proliferating cells express mRNAs with shortened 3' untranslated regions and fewer microRNA target sites. Science.

[9] Ji Z, Lee JY, Pan Z, Jiang B, Tian B (2009). Progressive lengthening of 3' untranslated regions of mRNAs by alternative polyadenylation during mouse embryonic development. Proc Natl Acad Sci U S A.

[10] Mayr C, Bartel DP (2009). Widespread shortening of 3'UTRs by alternative cleavage and polyadenylation activates oncogenes in cancer cells. Cell.

[11] Begik O, Oyken M, Cinkilli Alican T, Can T, Erson-Bensan AE (2017). Alternative Polyadenylation Patterns for Novel Gene Discovery and Classification in Cancer. Neoplasia.

[12] Zhang H, Sheng C, Yin Y, Wen S, Yang G, Cheng Z (2015). PABPC1 interacts with AGO2 and is responsible for the microRNA mediated gene silencing in high grade hepatocellular carcinoma. Cancer Lett.

[13] Mayr C (2017). Regulation by 3'-Untranslated Regions. Annu Rev Genet.

[14] Routh A, Ji P, Jaworski E, Xia Z, Li W, Wagner EJ (2017). Poly(A)-ClickSeq: click-chemistry for next-generation 3-end sequencing without RNA enrichment or fragmentation. Nucleic Acids Res.

[15] Gennarino VA, Alcott CE, Chen CA, Chaudhury A, Gillentine MA, Rosenfeld JA (2015). NUDT21-spanning CNVs lead to neuropsychiatric disease and altered MeCP2 abundance via alternative polyadenylation.

[16] Brumbaugh J, Di Stefano B, Wang X, Borkent M, Forouzmand E, Clowers KJ (2018). Nudt21 Controls Cell Fate by Connecting Alternative Polyadenylation to Chromatin Signaling. Cell.

[17] Tan S, Li H, Zhang W, Shao Y, Liu Y, Guan H (2018). NUDT21 negatively regulates PSMB2 and CXXC5 by alternative polyadenylation and contributes to hepatocellular carcinoma suppression. Oncogene.

[18] Hou T, Ou J, Zhao X, Huang X, Huang Y, Zhang Y (2014). MicroRNA-196a promotes cervical cancer proliferation through the regulation of FOXO1 and p27Kip1. Br J Cancer.

[19] Zhang N, Wei P, Gong A, Chiu WT, Lee HT, Colman H (2011). FoxM1 promotes beta-catenin nuclear localization and controls Wnt target-gene expression and glioma tumorigenesis. Cancer Cell.

[20] Sun M, Ding J, Li D, Yang G, Cheng Z, Zhu Q (2017). NUDT21 regulates 3'-UTR length and microRNA-mediated gene silencing in hepatocellular carcinoma. Cancer Lett.

[21] Wang W, Yang C, Nie H, Qiu X, Zhang L, Xiao Y (2019). LIMK2 acts as an oncogene in bladder cancer and its functional SNP in the microRNA-135a binding site affects bladder cancer risk. Int J Cancer.

[22] Hu H, Zhao J, Zhang M (2016). Expression of Annexin A2 and Its Correlation With Drug Resistance and Recurrence of Bladder Cancer. Technol Cancer Res Treat.

[23] Zhang L, Zhang W (2018). Knockdown of NUDT21 inhibits proliferation and promotes apoptosis of human K562 leukemia cells through ERK pathway. Cancer Manag Res.

[24] Christensen MV, Hogdall CK, Jochumsen KM, Hogdall EVS (2018). Annexin A2 and cancer: A systematic review. Int J Oncol.

[25] Liu Y, Li H, Ban Z, Nai M, Yang L, Chen Y (2017). Annexin A2 inhibition suppresses ovarian cancer progression via regulating beta-catenin/EMT. Oncol Rep.

[26] Wang Y, Chen K, Cai Y, Cai Y, Yuan X, Wang L (2017). Annexin A2 could enhance multidrug resistance by regulating NF-kappaB signaling pathway in pediatric neuroblastoma. J Exp Clin Cancer Res.

[27] Zhang Q, Zhao Z, Ma Y, Wang H, Ma J, He X (2014). Combined expression of S100A4 and Annexin A2 predicts disease progression and overall survival in patients with urothelial carcinoma. Urol Oncol.

[28] Zhang Y, Li A, Shi J, Fang Y, Gu C, Cai J (2018). Imbalanced LIMK1 and LIMK2 expression leads to human colorectal cancer progression and metastasis via promoting beta-catenin nuclear translocation. Cell Death Dis.

[29] Lou JC, Lan YL, Gao JX, Ma BB, Yang T, Yuan ZB (2017). Silencing NUDT21 Attenuates the Mesenchymal Identity of Glioblastoma Cells via the NF-kappaB Pathway. Front Mol Neurosci.

