# Supplementary material for: NUDT21 inhibits bladder cancer progression through ANXA2 and LIMK2 by alternative polyadenylation
Source: Theranostics. 2019 Sep 23;9(24):7156–67. doi: 10.7150/thno.36030 (PMC6831288; doi:10.7150/thno.36030)
Supplement: Supplementary file 1 — Supplementary figures and tables. [file thnov09p7156s1.pdf]

Supplemental Figure 1

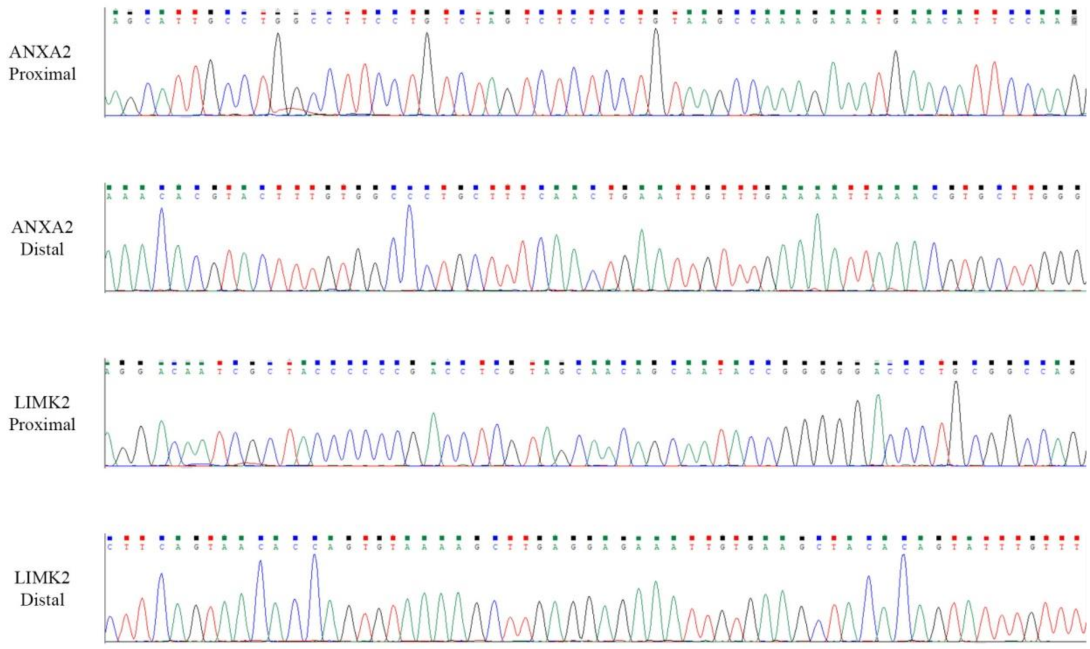

Supplemental Figure 2

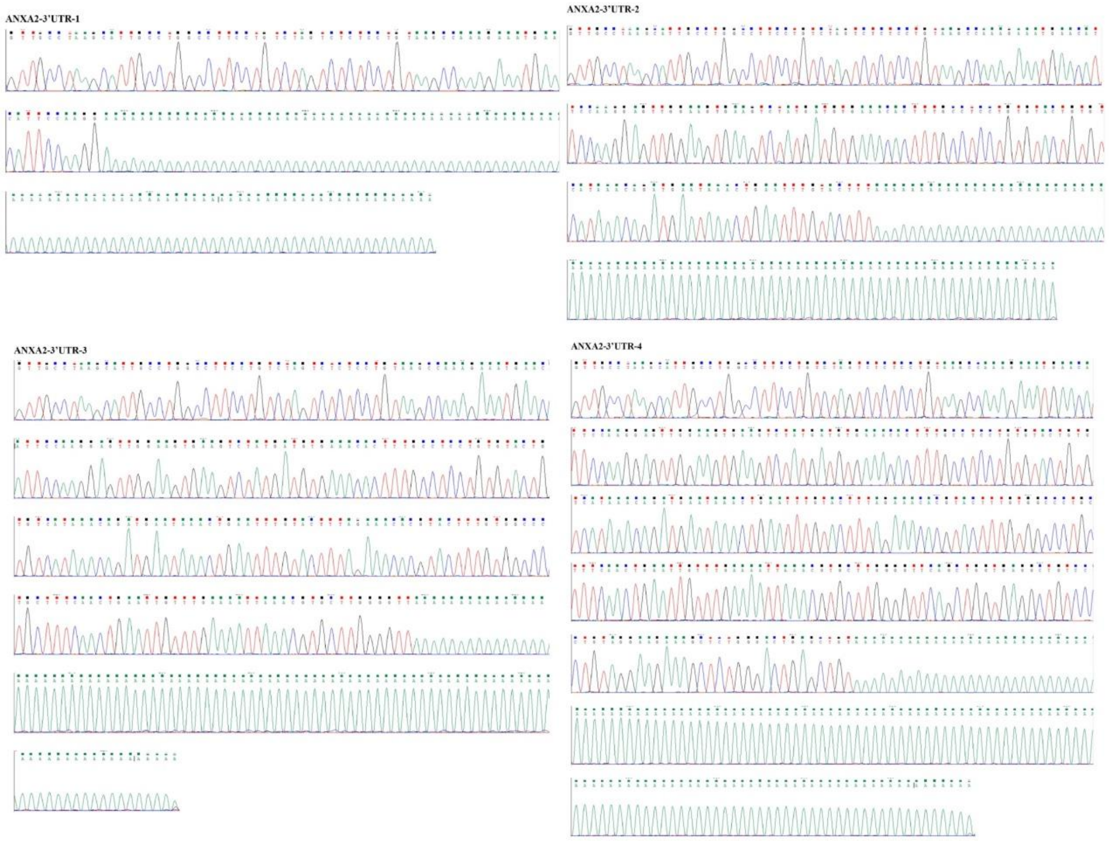

Supplemental Figure 3

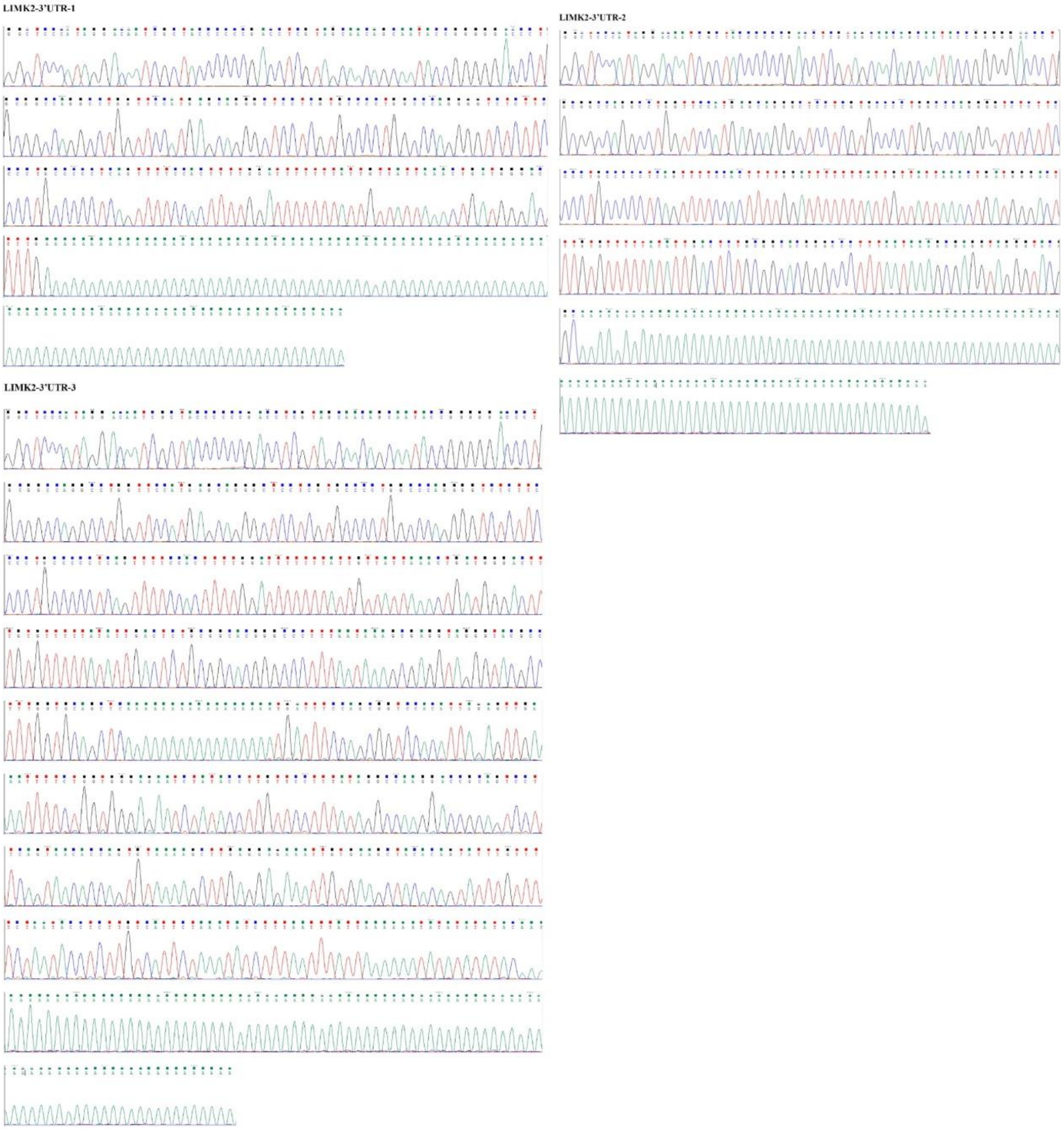

Supplemental Figure 4

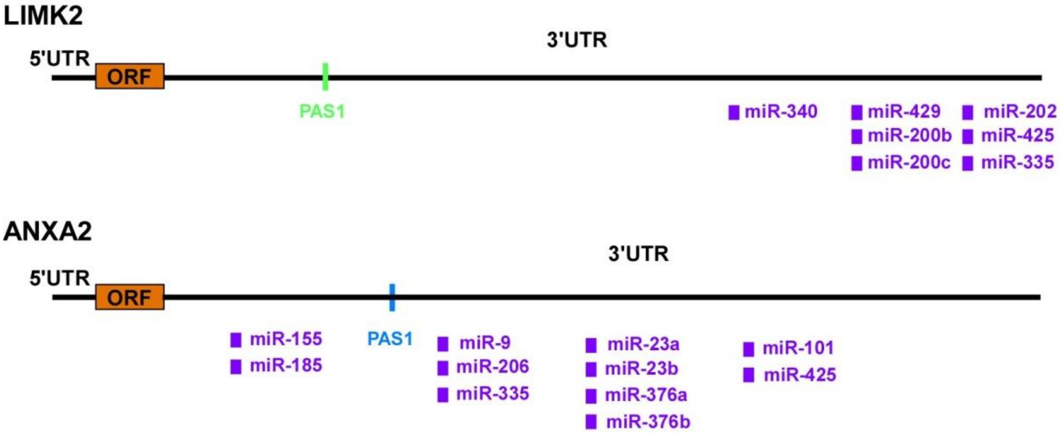

Supplemental table 1. Primers sequences.

Gene	Forward Primer (5′–3′)	Reverse Primer (5′–3′)
**mRNA real-time qPCR for figure 1D and 1F**		
NUDT21	TGCATTGGTAACTGGTGGAGAC	TGGGTCCATATCCTGGTGCATT
GAPDH	TCAAGAAGGTGGTGAAGCAG	CGTCAAAGGTGGAGGAGTG
**Cloning Primers for figure 4D**		
NUDT21-3'UTR-wt	CCGCTCGAGATTCCTGCGCA	AAATATGCGGCCGCTTCTCTTTCAA
NUDT21-3'UTR-mu	CTTGCCAGATGGGACTACTTAAGGAA	TTCCTTAAGTAGTCCCATCTGGCAAG
**PCR for figure 5B**		
CDK7 proximal	CATGTGGGCTGTTGGCTGTAT	CACTGTTCCTCAGTTGGTGTGC
CDK7 distal	TTGGGCACACCAACTGAGGAA	GCCGTAATTCGAGCACATGGA
CCBL2 proximal	TGACACACTCTGCATCAGCGA	TGTTCTCTCCCACATACCTGGA
CCBL2 distal	ACTCCACATAACCCACTTGGC	TTGTTCTCTCCCACATACCTGGA
PTPN18 proximal	CACACCCTGCTAGACTTCTGGA	GTCCTCATTCAGCCACTTCTCCT
PTPN18 distal	AAGTCTAACGCCAGTTCCTGC	TCCAGGCCCACTGTTTCCATT
ENSA proximal	CAAGAAGAAGAGAACCCTGCGG	CGAAGAACCTCCTTTAGCTCCAA
ENSA distal	CAAGAAGAAGAGAACCCTGCGGA	TTTGGCCTTTAGCTTTGCCTCT
UXS1 proximal	ACGTGTTGCAGAGACCATGTG	AAGTTGCTGACTACTCGCCCA
UXS1	TCAGTTTCTCTCCGAAGCCCA	TGGCGAGTCCGTCCTTTCTTT

distal		
ANXA2 proximal	ACACATCTGGTGACTTCCGCA	TTCCTCTTCACTCCAGCGTCA
ANXA2 distal	TTGCCTTCGCCTACCAGAGAA	AGCGTCATACTGAGCAGGTGT
EPB41L1 proximal	AAACCACGCCGAGACACAAG	CCGGTGGATGAGTTTGCTGTT
EPB41L1 distal	GCGTTCTTCCAGCAAACGGTA	CTTGTGTCTCGGCGTGGTTT
MTHFSD proximal	AGTGCTCGCTTCTTCGTCCTT	TCCCACCACAACTAAATCCACGA
MTHFSD distal	TGGTTCCAACACCACGACTGA	GTAGCCTTCTCCCTTCCCGATT
GATA3 proximal	AAATGAACGGACAGAACCGGC	TTGGCATTCCTCCTCCAGAGTG
GATA3 distal	AAATGAACGGACAGAACCGGC	TGTGGTTGTGGTGGTCTGACA
LIMK2 proximal	CGAACACTGGACTTTGGCCT	TCTAGGTTGAGGTGCTTCCGT
LIMK2 distal	ACTGTCAACGAAACCTGGCAC	ACCTTGCAGCTCATACAGGCA
MTHFD2 proximal	ATGGCTGCGACTTCTCTAATGTC	ACTTCCTGCTTGATCTGCTGGG
MTHFD2 distal	GCTGCGACTTCTCTAATGTCTGC	TTCTCGCCAACCAGGATCACA
MPPE1 proximal	GCCTGTGCTCAAAGCCATGTT	ACGCTCTCTCCATCTGCCATT
MPPE1	AGCTGTTGTCTTTGCTGTGCT	AACGCTCTCTCCATCTGCCATT

distal		
DNASE1L1 proximal	TTCTTTGGTTGGGCTTGTGCC	CCCACTTTGTCTCCACTCCTGA
DNASE1L1 distal	CAGCCTCACTGTTCTGTTGCTG	TCTAGGCACAAGCCCAACCAA
TFIP11 proximal	ACCTGGGTTGGAAGTCGATGT	TAGGCAATGTTCTCCCGTGCT
TFIP11 distal	TGACGAGCGGGAGAACTTTGA	AGTTGACTGGCGCAGAGTAGT

Gene	
**3'-RACE for figure 5C**	
ANXA2	GTTGCCTAAGCATTGCCTGG
LIMK2	GGCTCCCATAGGACAATCGC

Supplementary Table 2. Correlation between NUDT21 expression and clinicopathological features.

Feature	No.	NUDT21 expression		*P*
		Low	High	
Sex				0.233
Male	121	67	54	
Female	75	48	27	
Age (y)				0.995
≤60	92	54	38	
>60	104	61	43	

Tumor grade				**0.014**
Low	117	77	40	
High	79	38	41	
Tumor size				**0.024**
<3cm	143	77	66	
≥3cm	53	38	15	
T classification				**0.016**
Ta,T1	130	68	62	
T2-T4	66	47	19	
Total NO. of patients	196	115	81	

Supplementary Table 3. Multivariate analysis of overall survival (OS) and Recurrent-free survival (RFS) in patients with bladder cancer

Prognostic variables	OS		RFS	
	Hazard ratio (95% CI)	*P*	Hazard ratio (95% CI)	*P*
Sex (M vs F)	1.035 (0.547-1.957)	0.916	1.097 (0.695-1.732)	0.691
Age (>60 vs ≤60)	1.058 (0.584-1.916)	0.853	1.163 (0.778-1.738)	0.461
Tumor grade (High vs Low)	1.774 (0.941-3.344)	0.076	1.100 (0.699-1.733)	0.680
Tumor size (≥3cm vs<3cm)	1.758 (0.839-3.680)	0.135	1.685 (1.043-2.723)	**0.033**
T classification (T2-4 vs T1,Ta)	1.819 (1.015-3.262)	**0.045**	1.117 (0.721-1.733)	0.620
NUDT21 (High vs Low)	0.430 (0.224-0.826)	**0.011**	0.535 (0.352-0.815)	**0.004**
